# Different Impacts of Metabolic Syndrome Components on Insulin Resistance in Type 2 Diabetes

**DOI:** 10.1155/2013/740419

**Published:** 2013-01-31

**Authors:** Chung-Hua Hsu

**Affiliations:** ^1^Institute of Traditional Medicine, National Yang-Ming University, Taipei 112, Taiwan; ^2^Linsen (Chinese Medicine) Branch, Taipei City Hospital, Taipei, Taiwan

## Abstract

*Objective.* To examine the different impacts of MS components on insulin resistance in type 2 diabetes. *Methods.* A number of subjects (144) who met the criteria of (1) age between 30 and 75 years, (2) had type 2 diabetes for more than one year, and (3) taking gliclazide and metformin for more than 6 months were enrolled. All subjects were assigned to one of the four HOMA index categories. The HOMA index quartile 4 denotes the highest insulin resistance. The main outcome evaluated is the odds ratios (ORs) of different MS components on HOMA index quartile 4. The characteristics in HOMA index quartiles and groups of nonmetabolic syndrome (NMS; number of components < 2), metabolic syndrome A (MSA; number of components = 2), and metabolic syndrome B (MSB; number of components > 2) were also evaluated. *Results.* The results showed that both MSA and MSB groups had higher ORs (5.9 and 13.8 times, resp.) than the NMS group; and that subjects with large waist circumference (LWC) and high triglyceride (HTG) level have higher ORs (6.1 and 2.6 times, resp.) in developing higher insulin resistance than normal control subjects. *Conclusion.* Type 2 diabetic patients with greater number of MS components have higher ORs in developing increased insulin resistance.

## 1. Introduction

Insulin resistance is an important pathogenic factor in type 2 diabetic patients. Increased insulin resistance in type 2 diabetes is known to cause high risk in developing cardiovascular disease (CVD) [1–3]. Metabolic syndrome (MS) is an important cluster of metabolic abnormalities associated with insulin resistance and CVD [4, 5]. Despite the different definitions of MS proposed, high fasting plasma glucose level is one of the MS components [6, 7]. A high prevalence (35%–60%, depending on ethnicity, gender, and definition) of MS was found among type 2 diabetic patients [8–15]. Many studies have reported insulin resistance as the key pathogenic factor for the development of hyperlipidemia, glucose intolerance, hypertension, and obesity, all of which are MS components [16–23], while insulin resistance is not [6, 7]. Hence, the relationship between the components of MS and insulin resistance in type 2 diabetes merits further investigation.

Different severities or conditions of type 2 diabetes might lead to different degrees of insulin resistance. To understand the different impacts of MS components on insulin resistance in type 2 diabetes, and to avoid undetectable confounders and bias, this study enrolled subjects from a Chinese adult homogenous cohort aged between 30–75 years, who have had type 2 diabetes for more than one year and have been taking gliclazide and metformin for more than six months. 

We hypothesized that there would be different impacts of MS components on insulin resistance among type 2 diabetes after controlling for other factors in the homogenous diabetes cohort. This study also compared the characteristics among different numbers of MS components in this cohort.

## 2. Research Design and Method 

### 2.1. Study Population

The trial was conducted from July 2005 through June 2006 in Taipei Hospital, Taiwan. A total of 1356 registered diabetic patients were screened and 186 met the following criteria: (1) age between 30 and 75 years old, (2) being Chinese, (3) having been type 2 diabetes for more than one year, and (4) having been taking gliclazide and metformin for more than six months without taking other antidiabetic medication or insulin injection. The exclusion criteria include (1) GOT, GPT >80 U/L, serum creatinine >2.0 mg/dL, (2) lactation or pregnancy, (3) heart failure with New York Heart Association (NYHA) class >I, acute myocardial infarction, stroke, and that caused disabilities, and (4) any other conditions not suitable for trial as evaluated by the physician. After giving extensive oral and written information, a written informed consent form was obtained from the subjects before commencing any study-related activity. Finally, 144 subjects were enrolled in the study. The protocol was approved by the Human Ethics Committee of Taipei Hospital. 

### 2.2. MS Components

According to ATP III Asian (Chinese) definition, the components of MS are (1) large waist circumference (LWC) ≥80 cm in female and ≥90 cm in male, (2) high triglyceride (HTG) ≥150 mg/dL, (3) low HDL-cholesterol (LHDL) <40 mg in male and <50 mg in female, and (4) high blood pressure (HBP) ≥130/85 mg or on medication [6, 7].

### 2.3. Definition Groups of Non-MS (NMS), MS A (MSA), and MS B (MSB)

Subjects with a number of MS components <2 were classified as the NMS group, those with a number of MS components =2 were grouped under MSA, and those with a number of MS components >2 were grouped under MSB.

### 2.4. Assessment

Homeostasis model assessment for insulin resistance (HOMA index) (fasting glucose (mmol/L) × fasting insulin (UI/L)/22.5) was employed for evaluating insulin resistance [24, 25]. All subjects were assigned to one of the HOMA index categories according to the following quartiles: quartile 1 (<25%), quartile 2 (25%–49%), quartile 3 (50%–75%), and quartile 4 (>75%) for further assessment and comparison. The HOMA index quartile 4 denotes the highest insulin resistance. The main outcome evaluated is the ORs of different MS components on HOMA index quartile 4, which were assessed using multiple logistic regression analysis. The characteristics in HOMA index quartiles and groups of NMS, MSA, and MSB were also evaluated.

Anthropometric measurements including blood pressure, fasting glucose, HbA1C, insulin, adiponectin and triglyceride, cholesterol, cholesterol-HDL (HDL), and cholesterol-LDL (LDL) were taken. Waist circumference (WC) was measured midway between the lateral lower rib margin and the iliac crest, while hip circumference (HC) was measured at the levels of the major trochanters through the pubic symphysis. Height was measured with a wall-mounted stadiometer to the nearest 0.1 cm, weight was measured on a calibrated balance beam scale to the nearest 0.1 kg, and BMI was calculated (BMI = body weight/height (kg/m^2^)). A mercury sphygmomanometer with standard cuff was employed to measure the indirect auscultatory arterial blood pressure taken from the right arm with subjects in seated position. 

All measurements were made at 0800–0900 after an overnight fast using standardized methods. A sample of whole blood was drawn and centrifuged at 4°C, and a 1-mL aliquot of serum was rapidly frozen (−80°C) for subsequent hormone analysis. The plasma adiponectin concentration was measured by a radioimmunoassay kit (Linco Research, Inc., St. Charles, MO, USA). This kit employs the double-antibody/polyethylene glycol technique using ^125^I-lableled adiponectin and a multispecies adiponectin rabbit antiserum. Plasma insulin levels were measured using a commercially available radioimmunoassay (Linco Research Inc.). The intra- and interassay coefficients of variation were 3.1% and 4.9%, respectively. The limit of sensitivity is 0.5 ng/mL. 

### 2.5. Statistical Analysis

One-way analysis of variance (ANOVA) and linear trend test were employed to evaluate the trend among the HOMA index quartile groups and among groups of NMS, MSA, and MSB. Chi-square test was used for comparison of gender (male/female), family history of type 2 diabetes, and the percentage of MS component among groups. Multiple logistic regression analysis was employed for analysis among different MS components and groups of NMS, MSA, and MSB on the highest HOMA insulin resistance index quartile 4, with other factors adjusted. All *P* values were two-tailed and *α* level of significance was set at 0.05. The data were analyzed with SPSS software (version 11.5).

## 3. Results

### 3.1. Demographics

Among the 1356 screened patients, 186 met the inclusion criteria and 144 (77.4%) agreed to participate. There are 79 females (age 59.0 ± 8.7 years) and 65 males (age 56.3 ± 11.7 years). There is no significant gender difference in means of age, BMI, WC, blood pressure, HbA1C, fasting glucose, cholesterol, triglycerol, insulin, HOMA index, aminotransferases alanine, aminotransferases aspartate, and creatinine. 

### 3.2. Comparison among Quartiles of HOMA Index

Table 1 displays the comparison of characteristics among quartiles of HOMA index categories. As can be seen, the means of HOMA index quartile 4 and all the subjects are 9.3 (3.2) and 4.8 (3.3). Results of linear trend test show significant difference in %LWC (*P* < 0.001), %HTN (*P* = 0.006), %HTG (*P* = 0.001), BMI (*P* < 0.001), WC (*P* < 0.001), HC (*P* = 0.002), diastolic blood pressure (*P* = 0.002), fasting glucose (*P* < 0.001), HbA1C (*P* = 0.001), adiponectin level (*P* = 0.006), triglyceride level (*P* = 0.01), and cholesterol level (*P* = 0.03), but no statistical difference in family history of type 2 diabetes, gender ratio, %LHDL, and dose of metformin and gliclazide taken among quartiles of HOMA index.

### 3.3. Comparison among NMS, MSA, and MSB Groups

Table 2 displays the demographic and biochemical characteristics among the NMS, MSA, and MSB groups. As can be seen, 64.6% of the subjects met the ATP III Asian (Chinese) definition of having MS (MSA 36.2% and MSB 28.5%). Results of linear trend test show significant difference in means of HOMA index, BMI, WC, HC, blood pressure, percentage of all MS components, and insulin level (all the above having *P* < 0.001), but no statistical difference in family history of type 2 diabetes, HbA1C, and dose of metformin and gliclazide taken among the three groups.

### 3.4. Odds Ratio of Multiple Logistic Regression on HOMA Index Quartile 4 (The Highest Insulin Resistance)

Table 3 shows the ORs of different MS groups, gender, family history of type 2 diabetes, different BMI categories, and HbA1C groups on the highest HOMA index group (quartile 4), after adjustment of age, gender, family history of type 2 diabetes, and dose of metformin and gliclazide taken. The results showed that both MSA and MSB groups had higher ORs (5.9 and 13.8 times, resp.) than the NMS group, obese subjects (BMI ≥ 27) had higher ORs (2.8 times) than nonobese subjects, and high HbA1C subjects (>9.0) had higher ORs (3.6 times) than control groups in developing higher HOMA index.  Table 4 shows the ORs of different MS components (LWC, HTN, HTG, and LHDL), gender, family history of type 2 diabetes, different BMI categories, and HbA1C groups on the poor HOMA index group (quartile 4), after adjusting the age and dose of metformin and gliclazide taken. Significant difference can be seen in subjects with LWC, HTG, and high HbA1C having higher ORs, but not those with HTN and LHDL.

## 4. Discussion

Our initial findings evidenced different impacts of MS components on insulin resistance in type 2 diabetes. Subjects with LWC and HTG have higher ORs (6.1 and 2.6 times, resp.) in developing higher insulin resistance than subjects with normal WC and TG. The results also reveal that subjects with greater number of MS components have higher ORs in developing higher insulin resistance than the NMS group in this type 2 diabetic cohort.

Different severity or condition of type 2 diabetes might result in different insulin resistance, and taking different oral hypoglycemia agents might also influence insulin sensitivity [26–29]. Hence, we enrolled a homogenous adult Chinese cohort of 30–75 years old with type 2 diabetes for more than one year and having been taking gliclazide and metformin for more than six months to avoid undetectable confounders and bias.

Reaven reported insulin resistance as the central pathophysiological feature of the cluster of metabolic abnormalities, which has been associated with MS in many subsequent reports [4]. Many evidences also support the association between insulin resistance and vascular disease, and this has led to wide acceptance of the clustering of hyperlipidemia, glucose intolerance, hypertension, and obesity, all of which are MS components [30, 31]. Since there is still intense controversies among the definition of metabolic syndrome by ATPIII and its value as a CVD risk marker [32], we tried to figure out different impacts of the clustering MS components on insulin resistance of type 2 diabetic patients which has been proven to cause elevated risk of atherosclerosis [33]. 

Our results have reconfirmed the strong positive relationship between HOMA index and WC, BMI, HC, HbA1C, yes % of LWC, HTN HTG, and serum levels of glucose, triglyceride, and cholesterol (Table 1). All the above variables are the risk factors of developing complications in type 2 diabetes. It is interesting that yes % of LHDL, although being an MS component, shows no statistical significance in the trend test. The data have also demonstrated a negative relationship between the level of adiponectin and HOMA index in this homogenous type 2 diabetes cohort. Many studies have reported that adiponectin has both antiatherogenic and antidiabetic properties [34–36]. Previous research has shown that adiponectin levels are significantly lower in type 2 diabetes [3, 37, 38]. Subjects with hypoadiponectin level might have higher risk of developing cardiovascular disease [39, 40]. Adiponectin levels are positively correlated with insulin sensitivity and negatively with insulin resistance [34, 36, 40]. The findings in this study support that high HOMA index of type 2 diabetes has lower adiponectin level, which might lead to higher risk of developing cardiovascular disease in the future. 

Our data also revealed that both MSA and MSB groups have higher ORs in developing higher insulin resistance than the NMS group in this type 2 diabetes cohort. Moreover, subjects with greater number of MS components have higher ORs in developing higher insulin resistance (Table 3). Although insulin resistance is not an MS component, the above finding has reconfirmed the association between insulin resistance and the number of MS components in type 2 diabetes, which corresponds to the observation of Hsieh et al. in the early onset DM population [41]. We have a reason to believe that the greater the number of MS components in type 2 diabetes, the higher the risk in developing increased insulin resistance will be, and both of them lead to higher risk of CVD.

The finding in Table 4 also showed that groups with higher HbA1C (≥9.0) have higher ORs (3.6 times) in developing higher insulin resistance than the control group. Difference in BMI did not reach statistical significance. This may be because BMI can be fully predicted by waist circumference in this type 2 diabetes cohort. Previous studies have demonstrated that WC can be employed to predict insulin resistance [42], and some studies showed that WC is a better predictor of risk factors than BMI [43, 44]. Changes in BMI can be due to changes in skeletal muscle rather than body fat, whereas variations in WC are attributed mainly to changes in abdominal fat. Tankó et al. demonstrated that central abdominal fat is positively associated with insulin resistance [45]. According to previous studies, lipid and fatty acids may induce insulin resistance by blunting insulin sensitivity through inhibition of glycolysis at key points [46]. This explains the correlation of obesity, hyperlipidemia, and insulin resistance found in our study. From the results, we have a reason to believe that central obesity, hypertriglyceride, and long-term glucose control are the most important factors associated with insulin resistance in type 2 diabetes.

In conclusion, this study indicates the different impacts of MS components on insulin resistance in type 2 diabetes. Type 2 diabetic patients with greater number of MS components have higher ORs in developing higher insulin resistance. Central obesity and hypertriglyceride are the most important MS components associated with insulin resistance in type 2 diabetes.

## Figures and Tables

**Table 1:** Comparison of characteristics among quartiles of HOMA insulin resistance index.

Variable	Quartiles of HOMA insulin resistance index				*P* for trend
	1 Mean (SD)	2 Mean (SD)	3 Mean (SD)	4 Mean (SD)	
Basic data					
HOMA IR*	1.7 (0.7)	3.2 (0.4)	4.9 (0.7)	9.3 (3.2)	<0.001
Male/female	19/17	12/24	19/17	15/21	0.55
Age, year	60.5 (8.9)	60.5 (9.3)	56.4 (10.0)	54.3 (11.8)	0.003
FH*, yes/no	21/15	19/17	18/18	17/18	0.45
G*, mg/day	166.9 (45.0)	182.2 (41.1)	175.6 (46.1)	173.7 (30.6)	0.66
M*, mg/day	1648.6 (628.9)	1779.2 (601.7)	1784.7 (611.8)	1577.1 (583.8)	0.65
BMI*, kg/m^2^	24.8 (2.9)	24.8 (2.8)	26.6 (5.3)	28.5 (4.5)	<0.001
WC*, cm	83.6 (7.6)	83.4 (7.8)	87.3 (9.1)	93.0 (10.4)	<0.001
HC*, cm	93.8 (5.9)	93.9 (6.9)	96.2 (8.6)	99.8 (8.7)	0.002
SBP*, mmHg	131.9 (16.0)	135.9 (18.8)	136.3 (15.6)	139.3 (13.0)	0.07
DBP*, mmHg	77.4 (7.8)	79.5 (10.9)	80.8 (9.6)	80.5 (9.7)	0.002
MSC, yes %					
LWC	42.9	44.4	50.0	85.7	<0.001
HTN	14.3	30.6	33.3	45.7	0.006
HTG	25.7	36.1	44.4	62.9	0.001
LHDL	51.4	75.0	58.3	65.7	0.484
Fasting serum factors					
Glucose, mg/dL	169.3 (57.2)	184.5 (48.6)	198.2 (47.5)	223.6 (63.0)	<0.001
HbA1c, %	8.7 (1.5)	8.7 (1.6)	9.4 (1.8)	9.8 (1.3)	0.001
Insulin, IU/mL	4.6 (2.5)	7.4 (1.9)	10.8 (4.0)	17.5 (5.9)	<0.001
Adiponectin, *μ*g/mL	19.1 (7.7)	18.0 (7.0)	15.3 (7.7)	15.0 (6.2)	0.006
Fasting lipoprotein					
Triglyceride, mg/dL	130.9 (85.3)	155.7 (95.3)	189.3 (223.8)	270.4 (404.6)	0.01
Cholesterol, mg/dL	172.8 (33.9)	174.9 (40.1)	171.8 (30.3)	194.9 (48.4)	0.03
HDL, mg/dL	43.7 (8.9)	45.2 (9.8)	41.9 (8.3)	42.8 (9.6)	0.40
LDL, mg/dL	111.8 (30.8)	108.4 (33.2)	106.8 (30.9)	121.8 (36.6)	0.25

HOMA IR: HOMA insulin resistance index; FH: family history of type 2 diabetes disease; G: gliclazide; M: metformin; BMI: body mass index; WC: waist circumference; HC: hip circumference; SBP: systolic blood pressure; DBP: diastolic blood pressure; LWC: large waist circumference; HTN: high blood pressure; HTG: high triglyceride; LHDL: low HDL-cholesterol; HDL: HDL-cholesterol; LDL: LDL-cholesterol.

**Table 2:** Comparison of characteristics among the groups of nonmetabolic syndrome (NMS), metabolic syndrome A (MSA), and metabolic syndrome B (MSB).

Variable	NMS (components < 2) *n* = 51	MSA (components = 2) *n* = 52	MSB (components > 2) *n* = 41	*P* for trend
Basic data				
HOMA IR*	3.2 (1.0)	5.3 (4.0)	6.1 (3.0)	<0.001
Male/female	29/22	19/33	17/24	0.10
Age, year	60.9 (10.0)	56.4 (9.4)	55.8 (10.9)	0.01
FH*, %	58.8	44.2	56.1	0.29
G*, mg/day	171.0 (39.3)	175.4 (42.0)	175.6 (44.6)	0.59
M*, mg/day	1731.4 (566)	1763.5 (505)	1775.6 (727.1)	0.24
BMI*, kg/m^2^	24.2 (2.7)	27.0 (4.3)	27.7 (4.9)	<0.001
WC*, cm	82.0 (7.0)	87.7 (10.9)	92.0 (10.5)	<0.001
HC*, cm	91.8 (5.9)	97.5 (10.0)	99.2 (8.4)	<0.001
SBP*, mmHg	130.1 (13.2)	135.6 (18.2)	143.4 (13.3)	<0.001
DBP*, mmHg	75.6 (7.1)	80.7 (9.9)	86.5 (8.7)	<0.001
MSC, yes %				
LWC	20.0	71.2	80.5	<0.001
HTN	7.8	30.8	61.0	<0.001
HTG	7.8	38.8	90.2	<0.001
LHDL	43.1	59.6	87.8	<0.001
Fasting serum factors				
Glucose, mg/dL	185.5 (54.5)	188.4 (62.9)	211.1 (51.3)	0.04
HbA1c, %	9.1 (1.8)	8.8 (1.5)	9.7 (1.5)	0.07
Insulin, IU/mL	7.3 (4.5)	11.3 (6.8)	11.9 (6.1)	<0.001
Adiponectin, *μ*g/mL	18.2 (7.3)	17.8 (8.4)	14.0 (4.8)	0.01
Fasting lipoprotein				
Triglyceride, mg/dL	107.1 (60.8)	176.7 (190.4)	297.5 (369.1)	<0.001
Cholesterol, mg/dL	171.8 (38.4)	171.0 (32.4)	193.9 (45.5)	0.01
HDL, mg/dL	44.4 (10.0)	43.7 (10.2)	41.3 (6.3)	0.12
LDL, mg/dL	114.8 (29.4)	105.0 (32.2)	115.5 (38.5)	0.99

NMS: nonmetabolic syndrome; MSA: metabolic syndrome A; MSB: metabolic syndrome B; HOMA IR: HOMA insulin resistance index; FH: family history of type 2 diabetes disease; G: gliclazide; M: metformin; BMI: body mass index; WC: waist circumference; HC: hip circumference; SBP: systolic blood pressure; DBP: diastolic blood pressure; LWC: large waist circumference; HTN: high blood pressure; HTG: high triglyceride; LHDL: low HDL-cholesterol; HDL: HDL-cholesterol; LDL: LDL-cholesterol.

**Table 3:** Multiple logistic regression analysis for nonmetabolic syndrome (NMS), metabolic syndrome A (MSA), and metabolic syndrome B (MSB) on poor HOMA insulin resistance index quartile 4 (>75%).

Variables	Odds ratio	95% confidence interval
Metabolic syndrome (MS)		
NMS (components < 2)	Control	
MSA (components = 2)	5.9	1.1–31.5
MSB (components > 2)	13.8	2.7–69.8
Other factors		
Gender		
Female	Control	
Male	0.8	0.3–2.0
Family history of type 2 diabetes		
No	Control	
Yes	0.6	0.3–2.0
Obese, body mass index, kg/m^2^		
<27	Control	
≥27	2.8	1.0–7.7
HbA1c, %		
<9.0	Control	
≥9.0	3.6	1.3–9.6

The multiple logistic regression analysis, the dependent variable: poor HOMA insulin resistance index quartile 4 (>75%) was “yes” and the other quartiles were “no,” adjustment of age and dose of metformin and gliclazide taken.

**Table 4:** Multiple logistic regression analysis for metabolic syndrome factors and others factor on high HOMA insulin resistance index quartile 4 (>75%).

Variables	Odds ratio	95% confidence interval
Metabolic syndrome component		
Large waist circumfluence (LWC), cm		
Male < 90, female < 80	Control	
Male ≥ 90, female ≥ 80	6.1	1.7–22.7
Hypertension (HTN), mmHg		
<130/85	Control	
≥130/85	1.7	0.6–4.7
Hypertriglyceride (HTG), mg/dL		
<150	Control	
≥150	2.6	1.0–6.5
Low HDL-cholesterol (LHDL), mg/dL		
Male < 40, female < 50	Control	
Male ≥ 40, female ≥ 50	1.2	0.4–3.1
Other factors		
Gender		
Female	Control	
Male	0.9	0.3–2.5
Family history of type 2 diabetes		
No	Control	
Yes	0.8	0.3–2.0
Obese, body mass index, kg/m^2^		
<27	Control	
≥27	2.0	0.7–6.2
HbA1c, %		
<9.0	Control	
≥9.0	3.6	1.3–9.8

The multiple logistic regression analysis, the dependent variable: Poor HOMA insulin resistance index quartile 4 (>75%) was “yes” and the other quartiles were “no,” adjustment of age and dose of metformin and gliclazide taken.

## Abbreviations

HOMA index: HOMA insulin resistance index
FH: Family history of type 2 diabetes disease
G: Gliclazide
M: Metformin
BMI: Body mass index
WC: Waist circumference
HC: Hip circumference
SBP: Systolic blood pressure
DBP: Diastolic blood pressure
MS: Metabolic syndrome
MSA: Group of metabolic syndrome A
MSB: Group of metabolic syndrome B
NMS: Group of nonmetabolic syndrome
LWC: Large waist circumference
LTN: High blood pressure
HTG: High triglyceride
LHDL: Low HDL-cholesterol
HDL: HDL-cholesterol
LDL: LDL-cholesterol
ORs: Odds ratio.
